# Prognostic impact of body composition and immune-nutritional status in oligometastatic NSCLC patients receiving radiotherapy

**DOI:** 10.3389/fnut.2025.1588391

**Published:** 2025-07-01

**Authors:** Zhifei Huang, Ziwen Guo, Bo Gao, Shun Li, Yaner Yu, Shuangqiu Zhu, Zelai He, Haiyan Chen, Hao Jiang

**Affiliations:** ^1^Department of Radiation Oncology, The First Affiliated Hospital of Bengbu Medical University, Bengbu, Anhui, China; ^2^Joint Research Center for Regional Diseases of IHM, Bengbu Medical University, Bengbu, Anhui, China; ^3^Joint Research Center for Regional Diseases of IHM, The First Affiliated Hospital of Bengbu Medical University, Bengbu, Anhui, China; ^4^Anhui Provincial Key Laboratory of Tumor Evolution and Intelligent Diagnosis and Treatment, Bengbu Medical University, Bengbu, Anhui, China; ^5^Department of Radiation Oncology (Key Laboratory of Cancer Prevention and Intervention, China National Ministry of Education, Key Laboratory of Molecular Biology in Medical Sciences, Zhejiang, China), The Second Affiliated Hospital, Zhejiang University School of Medicine, Hangzhou, Zhejiang, China; ^6^Anhui Hospital, The Second Affiliated Hospital of Zhejiang University School of Medicine, Bengbu, Anhui, China

**Keywords:** body composition, immune-nutritional scores, oligometastasis, NSCLC, radiotherapy

## Abstract

**Background:**

Metabolic and nutritional status are recognized as prognostic and predictive biomarkers in cancer treatment. However, there is limited research on oligometastatic non-small cell lung cancer (NSCLC) patients undergoing radiotherapy. We aimed to explore the independent and synergistic effects of body composition and immune-nutritional status on survival outcomes in these patients.

**Methods:**

Patients with oligometastatic NSCLC who underwent radiotherapy between 2017 and 2022 were retrospectively included. The evaluated outcomes were overall survival (OS) and progression-free survival (PFS). The skeletal muscle index (SMI), subcutaneous fat index (SFI), and pericardial fat index (PFI) were obtained using computed tomography (CT) and normalized by height squared. Laboratory biomarkers were utilized to assess immune-nutritional status. Univariate chi-square tests and multivariate logistic regression analyses were employed to determine correlations between hematological parameters and body composition. We conducted K-M analyses, along with univariate and multivariate Cox regression analyses to evaluate survival outcomes.

**Results:**

102 patients [mean age 61.44 years, 51 males (50%)] were included. Compared to non-responders, responders exhibited a significantly lower prevalence of sarcopenia (44.93 vs. 55.07%, *P* = 0.007) and demonstrated relatively better immune-nutritional scores. Logistic regression analyses revealed that a low Prognostic Nutritional Index (PNI) was a risk factor for SMI, SFI, and PFI. Multivariate Cox analyses revealed that C reactive protein-to-albumin ratio (CAR) (HR = 0.26; 95% CI 0.11–0.61; *P* = 0.002) and PFI (HR = 2.73; 95% CI 1.06–7.01; *P* = 0.037) were predictive factors for OS. CAR (HR = 0.34, *P* = 0.001) and SFI (HR = 1.82, *P* = 0.049) were independent prognostic markers for PFS. K-M analyses indicated that the group with high PNI and high SFI exhibited markedly improved OS and PFS, as well as the one with high PNI and without sarcopenia (*P* < 0.001).

**Conclusion:**

Among oligometastatic NSCLC patients receiving radiotherapy, higher skeletal muscle and fat content, along with better immune-nutritional status, correlated with improved survival outcomes.

## 1 Introduction

Lung cancer remains a leading cause for cancer-related mortality globally, with non-small cell lung cancer (NSCLC) accounting for about 85% of diagnoses (1). Unfortunately, most NSCLC patients are diagnosed at advanced stages, frequently accompanied by metastases, resulting in poor prognoses (2). Oligometastatic NSCLC represents a specific subtype, characterized by limited and organ-site-specific metastasis, suggesting that enhancing standard treatment with targeted local therapies may improve survival outcomes (3). A randomized controlled trial has demonstrated that radiotherapy significantly improves overall survival (OS) compared to standard palliative care alone (4). However, predictive markers for therapeutic efficacy remain largely unexplored.

Recent studies have highlighted a significant correlation between body composition and cancer prognosis, particularly regarding skeletal muscle and adipose tissue (5–7). Some longitudinal studies indicate that in cancer patients, obesity serves as an independent risk factor for unfavorable prognosis, and elevated visceral fat is associated with disease progression and decreased postoperative survival (8–10). Additionally, sarcopenia is thought to be associated with poorer outcomes, as well as an increased risk of recurrence and metastasis (11). These impacts are believed to be related to immune and inflammatory responses (12). Infiltration of immune cells, including M1-polarized macrophages and T cells, causes metabolic inflammation in excess visceral adipose tissue, prompting adipose tissue to secrete pro-inflammatory cytokines. Such an inflammatory environment may impair the immune response in obese patients, exacerbating their prognosis (13). For example, as a marker of visceral fat, pericardial fat is linked to the prognosis of NSCLC (14). Moreover, macrophage infiltration within adipose tissue can initiate systemic inflammation, a hallmark of cancer. Systemic inflammatory response might promote catabolic pathways, contributing to muscle wasting in cancer patients (15). Numerous studies have demonstrated associations between systemic inflammatory markers and sarcopenia, emphasizing the profound impact of sarcopenia on survival outcomes, particularly when associated with an elevated systemic inflammatory response (SIR) (16–18). Additionally, a low skeletal muscle index (SMI), indicative of sarcopenia, is correlated with frailty and immobility in NSCLC patients (7).

Nonetheless, the influence of adipose tissue on survival remains contentious. Some studies report that visceral fat levels do not affect prognosis and may even show a positive impact on long-term survival (19). These discrepancies may arise from the heterogeneity of study populations, variations in data analysis methodologies, differing thresholds, and changes in clinical settings. Furthermore, few studies have investigated the impact of body composition and systemic inflammation in oligometastatic NSCLC patients receiving radiotherapy, and the generalizability of the existing conclusions across diverse populations remains underexplored. Currently, computed tomography (CT) provides a definitive and non-invasive method of quantifying skeletal muscle and adipose tissue (20). Therefore, we aimed to comprehensively analyze the impact of body composition and immune-nutritional status on survival of oligometastatic NSCLC patients undergoing radiotherapy, identify patients at risk, and promote more targeted and patient-centered treatment strategies.

## 2 Materials and methods

### 2.1 Patients

**Figure 1:**
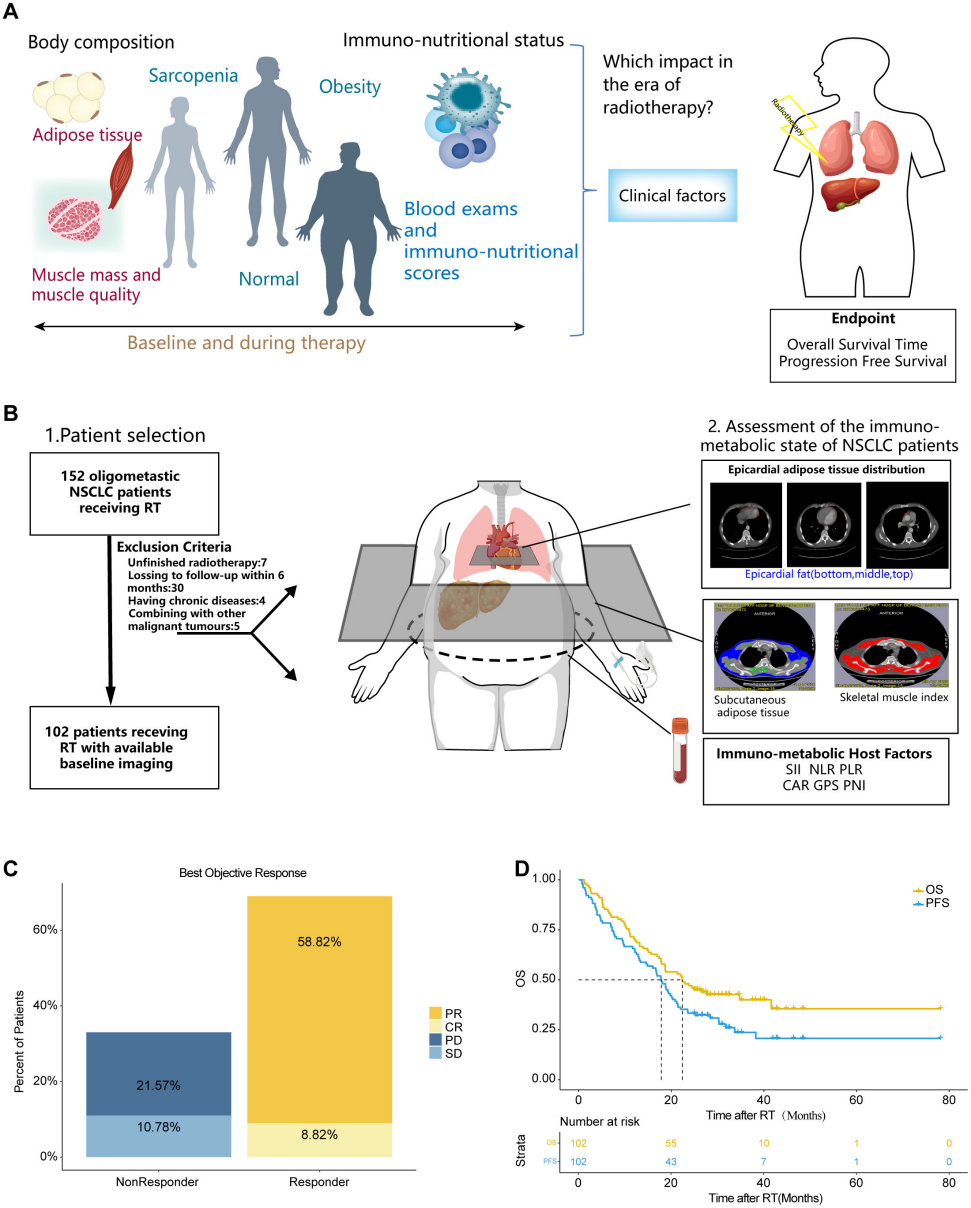
The study cohort demonstrates representative real-world clinical outcomes. **(A)** Graphical Abstract. **(B)** Schematic representation outlining from left to right: the study cohort with key exclusion criteria, methods for body composition measurements and assessment of the immuno-nutritional status, and the study endpoints. **(C)** Best objective response rate (ORR) at day 90, determined according to RECIST 1.1 criteria. **(D)** Kaplan-Meier estimates of PFS and OS for the entire study cohort. Median survival times are depicted in months. CR, complete remission; PR, partial remission; SD, stable disease; PD, progressive disease; OS, overall survival; PFS, progression free survival; SII, systemic immune-inflammation index; NLR, neutrophil-to-lymphocyte ratio; PLR, platelet-to-lymphocyte ratio; CAR, C reactive protein-to-albumin ratio; PNI, the Prognostic Nutritional Index; GPS, the Glasgow Prognostic Score; SFI, subcutaneous fat index; PFI, Pericardial Fat Index; SMI, Skeletal muscle index.

We retrospectively included patients at the First Affiliated Hospital of Bengbu Medical College who were diagnosed with oligometastatic NSCLC and underwent radiotherapy between December 2017 and December 2022. The inclusion criteria were the following: (a) a pathological diagnosis of oligometastatic NSCLC; (b) administration of radiotherapy; (c) comprehensive medical history and follow-up data; (d) accessible CT scans taken before treatment. After excluding patients who did not complete radiation therapy or suffered from chronic conditions such as hematological or autoimmune diseases, as well as those with other concurrent malignancies, 102 patients were included, and all had available baseline imaging (Figure 1). This retrospective study adhered to the Declaration of Helsinki and was approved by the Ethics Committee of the First Affiliated Hospital of Bengbu Medical University.

### 2.2 Data collection

We collected general information from the patients, including age, sex, BMI, the presence of chronic diseases, smoking history, tumor histological type, the number of metastatic organs, oligometastatic state, the Eastern Cooperative Oncology Group (ECOG) performance status and laboratory test indicators such as low-density lipoprotein (LDL), high-density lipoprotein (HDL), cholesterol, triglycerides, albumin, and C-reactive protein (CRP). Immuno-nutritional scores were derived from specific laboratory biomarkers. CRP and albumin levels were used to determine the Glasgow prognostic score (GPS) (21): a CRP ≤ 10 mg/dL scored 0; a CRP > 10 mg/dL with albumin > 3.5 g/dL scored 1; a CRP > 10 mg/dL with albumin < 3.5 g/dL scored 2. The C reactive protein-to-albumin ratio (CAR) was calculated by dividing CRP (mg/L) by albumin (g/dL) (22). The systemic immune-inflammation index (SII) was calculated as the product of neutrophil count and platelet count divided by lymphocyte count (10^9^/L) (23). The neutrophil-to-lymphocyte ratio (NLR) was determined by dividing the absolute neutrophil count by the total lymphocyte count (24). The prognostic nutritional index (PNI) was determined using the following equation: albumin (g/dL) plus 0.005 times the total lymphocyte count (25). The platelet-to-lymphocyte ratio (PLR) was calculated by dividing the platelet count (× 10^9^/L) by the lymphocyte count (× 10^9^/L) (23). Further details can be found in Supplementary Table 1. All these blood biomarkers reflecting metabolic or nutritional status were obtained within 14 days prior to the initiation of radiotherapy.

### 2.3 CT image analysis

Although skeletal muscle is typically measured at the third lumbar (L3) level using CT scans, chest CT is more commonly utilized in clinical practice, especially in regions like China, where CT lung cancer screening is routinely performed. Therefore, quantifying pectoralis central muscle using chest CT can provide a more effective method for routine body composition analysis, avoiding additional radiation exposure and complicated measurements (26).

We evaluated body composition characteristics based on the best response criteria derived from pre-lymphodepletion imaging studies. The outer margins of the bilateral pectoralis major and minor muscles on non-contrast chest CT images were manually delineated at the level of the fourth thoracic vertebra (Th4) by a radiologist with experience in chest imaging, who was unaware of patient information. The pre-processed scans were analyzed using sliceOmatic (Tomovision V.5.0) following established methods, and we obtained skeletal muscle area (cm^2^). From the same image, we measured the subcutaneous fat area (cm^2^) extending from the pectoral muscles to the pectoralis major. The Hounsfield unit threshold for skeletal muscle and subcutaneous fat were −29 to 150 and −190 to −30, respectively. Pericardial fat was identified as the area extending vertically from the right pulmonary artery to the diaphragm and horizontally from the left edge of the heart apex to the right edge of the atrium, using CT density within the range of −190 to −30 HU. Once pericardial fat was identified, its volume (cm3) was automatically calculated. Sarcopenia is assessed by normalizing the cross-sectional area of skeletal muscle (cm^2^) by height squared (m^2^), resulting in SMI (cm^2^/m^2^) (7). The subcutaneous fat index (SFI) (cm^2^/m^2^) was calculated by normalizing the subcutaneous fat area to height squared (m^2^) (27). The pericardial fat index (PFI) (cm3/m^2^) was derived by dividing the pericardial fat volume by body surface area using the Mosteller formula (14).

### 2.4 Endpoints

We define the time from the commencement of therapy until death as OS and the duration from the commencement of therapy to disease progression or death as Progression-Free Survival (PFS). Efficacy assessment was performed 90 days following the completion of radiotherapy, at which point optimal therapeutic outcomes are expected. We assessed the tumor response according to the RECIST 1.1 criteria, classifying responses as complete response (CR), partial response (PR), stable disease (SD), or progressive disease (PD). Patients who achieved PR or CR were categorized as responders, whereas those with SD or PD were non-responders.

### 2.5 Statistical analysis

We assessed normal distribution using the d'Agostino-Pearson test. Continuous variables were represented by their median and interquartile range (IQR), unless stated otherwise. Categorical variables were expressed as numbers with percentages. Comparisons of continuous variables between groups were conducted using the Mann-Whitney *U*-test or Student's *t*-test, while categorical variables were analyzed using Fisher's exact test or chi-square test. We used the Spearman test to evaluate correlations between parameters. Chi-square tests and logistic regression were used to clarify the associations between hematological parameters and body composition. Following univariate logistic regression analyses, variables yielding a *p*-value < 0.1 were selected for further multivariate logistic regression. Both univariate and multivariate Cox proportional hazard models were used to calculate hazard ratios (HRs) for mortality and to identify predictive factors for death outcomes. Cut-off values were established through a survival-based software package fitting a Cox proportional hazards model. Nonetheless, given the small sample sizes and the absence of statistical significance in most Cox proportional hazards models, we chose to use values previously reported in the literature or established normal laboratory values (PNI, NLR, PLR). K-M curves facilitated the comparison of survival distributions between groups using time-dependent tests. Two-sided *P*-values < 0.05 are considered statistically significant. R 4.1.3 was used to analyze all data.

## 3 Results

### 3.1 Patient characteristics

There are 102 patients with oligometastatic NSCLC, comprising half male participants. The average age of participants was 61.44 years. Histological examination revealed that adenocarcinoma was the predominant subtype (*n* = 67, 65.69%). 66.67% (*n* = 68) of the patients presented with single-organ metastases. 63.73% (*n* = 65) of the patients had hypertension, and 78.43% (*n* = 80) were diagnosed with diabetes mellitus. A total of 61 patients (59.8%) underwent radiotherapy targeting the brain, while 19 (18.63%) to bone, and 8 (7.84%) to lung. Other locations accounted for 13.73%. The ECOG performance status was significantly lower in responders (*P* < 0.001), and baseline CRP levels were markedly higher (5.83 vs. 2.8 mg/dL, *P* = 0.002) in non-responders. Detailed information is shown in Table 1. The overall response rate at 90 days post-radiotherapy was 67.64%, with a complete response rate of 8.82% (Figure 1C). After a median follow-up of 22.4 months, the median PFS (mPFS) and median OS (mOS) were 17.8 months and 22.37 months, respectively (Figure 1D).

**Table 1:** Baseline patient characteristics.

**Characteristic**	**All patients (*N* = 102)**	**Responders (*N* = 69)**	**Non-responders (*N* = 33)**	***P*-value**
**General characteristics**				
Age (mean ± sd)	61.44 ± 9.37	61.12 ± 8.65	62.13 ± 0.83	0.615
Sex (female), *n* (%)	51 (50.00)	33 (47.83)	18 (54.55)	0.672
Hypertension, *n* (%)	65 (63.73)	43 (62.32)	22 (66.67)	0.83
Hyperglycemia, *n* (%)	80 (78.43)	27 (81.82)	53 (76.81)	0.751
ECOG				**< 0.001**
≥2	43 (42.16)	10 (14.49)	33 (100.00)	
0–1	59 (57.84)	59 (85.51)	0 (0.00)	
Histological diagnoses				0.501
Adenocarcinoma	67 (65.69)	43 (62.32)	24 (72.73)	
Squamous cell carcinoma	34 (33.33)	25 (36.23)	9 (27.27)	
Metastasis location				0.764
Bone	19 (18.63)	11 (15.94)	8 (24.24)	
Brain	61 (59.80)	42 (60.87)	19 (57.58)	
Lung	8 (7.84)	6 (8.70)	2 (6.06)	
Others	14 (13.73)	10 (14.49)	4 (12.12)	
Number of metastic organ				0.501
Single	68 (66.67)	44 (63.77)	24 (72.73)	
Multiple	34 (33.33)	25 (36.23)	9 (27.27)	
Oligotransition state				**0.011**
Synchronous	48 (47.06)	39 (56.52)	9 (27.27)	
Metachronous	54 (52.94)	30 (43.48)	24 (72.73)	
**Laboratory biomedical features**				
LDL (mmol/L, mean ± sd)	2.767 ± 0.849	2.731 ± 0.88	2.843 ± 0.788	0.537
HDL [mmol/L, median (IQR)]	1.06 (0.91, 1.33)	1.09 (0.90, 1.36)	1.02 (0.92, 1.32)	0.724
Cholesterol [median (IQR)]	4.35 (3.78, 5.21)	4.33 (3.77, 5.14)	4.79 (3.80, 5.22)	0.543
Triglyceride [median (IQR)]	1.28 (0.98, 1.88)	1.39 (0.97, 1.91)	1.23 (1.01, 1.75)	0.852
Albumin [median (IQR)]	40.89 (38.05, 42.80)	41.00 (38.30, 42.80)	39.80 (37.8, 42.8)	0.442
CRP [median (IQR)]	3.51 (1.12, 14.47)	2.80 (0.90, 7.50)	5.83 (3.0, 31.6)	**0.002**

CRP, C reactive protein.

Values are shown as median (IQR) or unless number (percent) if not stated otherwise. *P*-values < 0.05 are highlighted in bold.

### 3.2 Responders exhibit sarcopenia and altered immuno-nutritional scores

Using CT imaging and blood biomarkers, we calculated and compared the body composition and immuno-nutritional scores of the participants (Table 2). A significant difference in BMI was observed between responders and non-responders (*P* < 0.05). Specifically, the prevalence of overweight individuals was higher among responders, while a greater proportion of underweight individuals was found in the non-responder group. Indicators assessing adipose and muscle tissue distribution, including SFI, PFI, did not reveal statistically significant differences. However, the prevalence of sarcopenia was markedly elevated among non-responders (75.76 vs. 44.93%, *P* = 0.006). Additionally, we examined established immuno-nutritional scores, such as GPS, CAR, and PNI. Responders exhibited lower GPS scores (GPS = 0: 68.12 vs. 45.45%, *P* = 0.048), reduced CAR (CAR > 1.44: 14.49 vs. 51.52%, *P* < 0.001), and higher PNI (PNI > 45: 84.06 vs. 48.48%, *P* < 0.001). Between the two groups, no significant differences were observed in the inflammatory markers, including NLR, PLR, and SII. These results suggested that sarcopenia, nutritional status, and inflammation may serve as prognostic indicators for early responses to radiotherapy.

**Table 2:** Distribution of body composition and immuno-nutritional status between responders and non-responders.

**Characteristic**	**All patients (*N* = 102)**	**Responders (*N* = 69)**	**Non-responders (*N* = 33)**	***P*-value**
**Nutritional status**				
**BMI categories**				***P*** **<** **0.001**
Normal	62 (60.78)	42 (60.87)	20 (60.61)	
Overweight	28 (27.45)	24 (34.78)	4 (12.12)	
Underweight	12 (11.76)	3 (4.35)	9 (27.27)	
**Adipose and muscle tissue distribution**				
SFI (Rf: SFI > 26.4)	61 (59.80)	45 (65.22)	16 (48.48)	0.163
PFI (Rf: PFI > 38.66)	26 (25.49)	20 (28.99)	6 (18.18)	0.353
**Sarcopenia (Rf: SMI** **<** **58.32)**	56 (54.90)	31 (44.93)	25 (75.76)	**0.006**
**Immuno-nutritional scores**				
PNI (Rf: PNI > 45)	74 (72.55)	58 (84.06)	16 (48.48)	***P*** **<** **0.001**
GPS (Rf: GPS = 0)	62 (60.78)	47 (68.12)	15 (45.45)	**0.048**
CAR (Rf: CAR > 1.44)	27 (26.47)	10 (14.49)	17 (51.52)	***P*** **<** **0.001**
**Inflammation indexes**				
SII (Rf: SII > 296.31)	78 (76.47	55 (79.71)	23 (69.70)	0.387
PLR (Rf: PLR > 169.1)	40 (39.22)	25 (36.23)	15 (45.45)	0.499
NLR (RF:NLR > 3.8)	42 (41.18)	25 (36.23)	17 (51.52)	0.210

Data are median (IQR) or *n* (%) unless otherwise stated.

Abbreviation: SFI, subcutaneous fat index; PFI, Pericardial Fat Index; SMI, Skeletal muscle index; PNI, the Prognostic Nutritional Index; GPS, the Glasgow Prognostic Score; CAR, C-reactive protein-to-albumin ratio; SII, systemic immune-inflammation index; PLR, platelet-to-lymphocyte ratio; NLR, neutrophil-to-lymphocyte ratio.

### 3.3 Body composition and prognosis nutritional index are predictors for survival in oligometastatic NSCLC receiving radiotherapy

**Figure 2:**
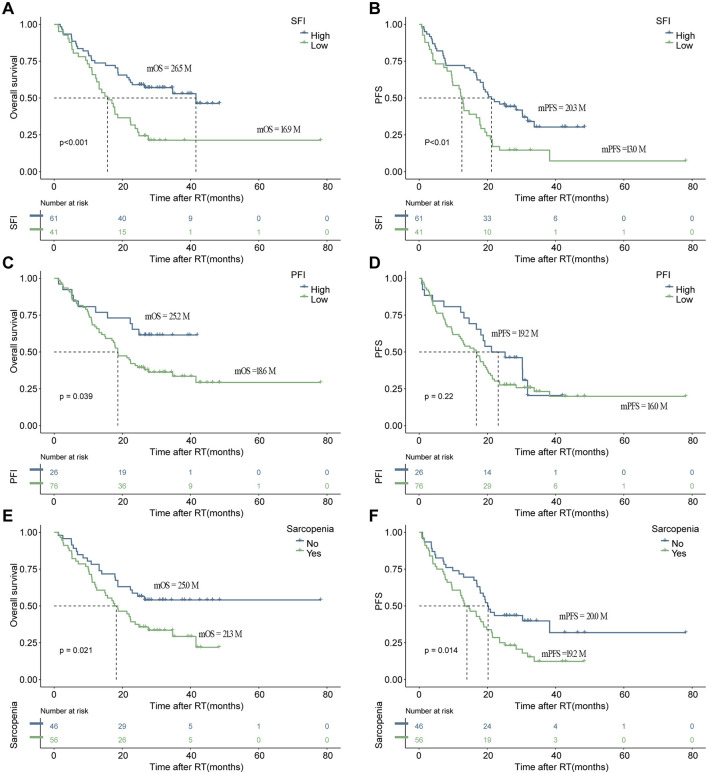
The depicts the association of increased body composition with superior progression-free survival (PFS) and overall survival (OS). Kaplan–Meier estimates show median OS (left) and PFS (right) stratified by SFI **(A, B)**, and PFI **(C, D)**, as well as sarcopenia **(E, F)**. The respective cutoff for each parameter and median survival in months are indicated above each graph. The *P*-value from the Mantel–Cox log-rank test is provided in the graph inset. SFI, subcutaneous fat index; PFI, Pericardial Fat Index.

**Figure 3:**
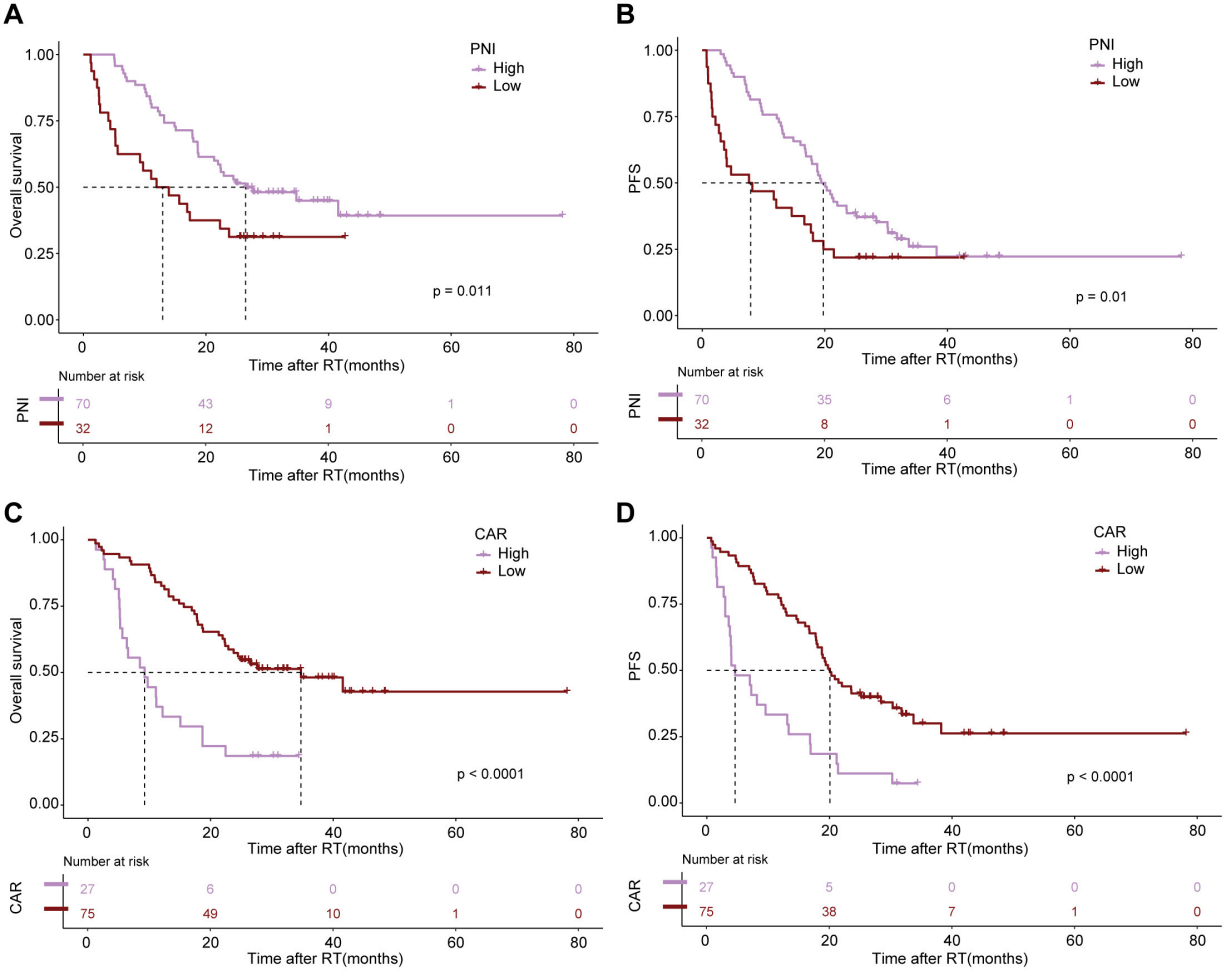
The impact of altered immuno-nutritional scores as negative prognostic markers for survival outcomes. Kaplan–Meier estimates display median OS (left) and PFS (right) stratified by Prognostic Nutritional Index [PNI; **(A, B)**] and CRP-to-Albumin ratio [CAR; **(C, D)**]. The respective cutoff for each parameter and median survival in months are depicted above each graph. The *P*-value from the Mantel–Cox log-rank test is indicated in the graph inset.

After conducting cutoff analyses, we delineated high and low-risk groups according to body composition markers (PFI, SFI, SMI) and immuno-nutritional scores (CAR, PNI, SII, GPS, NLR, PLR). In the univariate Cox regression analysis, ECOG performance status, BMI, PFI, SFI, sarcopenia, PNI, and CAR were significantly linked to OS (*P* < 0.05). ECOG performance status, underweight status, CAR, and PFI emerged as independent prognostic markers (*P* < 0.05), following inclusion in multivariate Cox regression analysis (Table 3). For PFS, ECOG performance status, albumin levels, BMI, SFI, sarcopenia, PNI, and CAR demonstrated significant correlations (*P* < 0.05). In the multivariate Cox regression analysis, ECOG performance status, underweight status, SFI, and CAR were identified as significant predictors of PFS (*P* < 0.05) (Table 4). K-M analyses revealed significant differences in OS between high and low-risk groups stratified by SFI, and PFI (*P* < 0.05). Notably, significant differences in PFS were observed only between high and low-risk groups based on SFI (*P* < 0.05). SFI, and PFI were associated with comparatively better prognoses (Figure 2). Among patients without sarcopenia, both OS and PFS were markedly improved (*P* < 0.05) (Figure 2). Furthermore, significant differences in OS and PFS were noted between groups stratified by PNI and CAR (*P* < 0.05), with higher PNI and lower CAR indicating relatively better prognostic outcomes (Figure 3).

**Table 3:** Univariable and multivariable Cox regression for OS.

**Characteristic**	**Univariable**		**Multivariable**	
	**HR (95% CI)**	***P-*value**	**HR (95% CI)**	***P-*value**
**Demographic features**				
Age	1.02 (0.99–1.05)	0.242		
Sex (Rf = male)	0.83 (0.5–1.38)	0.479		
ECOG (Rf = 0–1)	0.25 (0.15–0.42)	**< 0.001**	0.3 (0.14–0.61)	**0.001**
Hypertension (Rf = yes)	1.14 (0.67–1.91)	0.633		
Hyperglycemia (Rf = yes)	0.71 (0.36–1.39)	0.318		
**Laboratory biomedical features**				
HDL	0.89 (0.47–1.66)	0.712		
LDL	0.94 (0.7–1.27)	0.694		
Cholesterol	0.98 (0.79–1.23)	0.865		
Triglyceride	1.05 (0.83–1.33)	0.659		
Albumin	0.96 (0.92–1.00)	0.066		
**Anthrometric measures**				
**BMI categories, (kg/m** ^2^ **)**				
Underweight	3.93 (1.98–7.78)	**< 0.001**	6.42 (2.19–18.87)	**< 0.001**
Overweight	0.41 (0.21–0.84)	**0.014**	0.54 (0.24–1.23)	0.143
**Adipose and muscle tissue distribution**				
SFI (Rf: SFI < 26.4)	2.33 (1.39–3.9)	**0.001**	1.54 (0.79–2.99)	0.201
PFI (Rf: PFI < 38.66)	2.02 (1.02–3.99)	**0.043**	2.73 (1.06–7.01)	**0.037**
**Sarcopenia (Rf: Without sarcopenia)**	1.85 (1.09–3.15)	**0.023**	0.83 (0.41–1.68)	0.601
**Immuno-nutritional scores**				
PNI (Rf: PNI < 45)	1.96 (1.16–3.33)	**0.012**	0.80 (0.40–1.62)	0.538
GPS (Rf: GPS = 1–2)	1.48 (0.89–2.47)	0.133		
CAR (Rf: CAR < 1.44)	0.31 (0.18–0.54)	**0.001**	0.26 (0.11 - 0.61)	**0.002**
**Inflammation indexes**				
NLR (RF:NLR < 3.8)	0.89 (0.53–1.48)	0.643		
SII (Rf: SII < 296.31)	1.66 (0.96–2.9)	0.072		
PLR (Rf: PLR < 169.1)	1.04 (0.62–1.76)	0.872		

Abbreviation: SFI, subcutaneous fat index; PFI, Pericardial Fat Index; SMI, Skeletal muscle index; PNI, the Prognostic Nutritional Index; GPS, the Glasgow Prognostic Score; CAR, C-reactive protein-to-albumin ratio; SII, systemic immune-inflammation index; PLR, platelet-to-lymphocyte ratio; NLR, neutrophil-to-lymphocyte ratio; HR, hazard ratio; CI, confidence interval.

**Table 4:** Univariable and multivariable Cox regression for PFS.

**Characteristic**	**Univariable**		**Multivariable**	
	**HR (95% CI)**	***P*-value**	**HR (95% CI)**	***P*-value**
**Demographic features**				
Age	1.02 (0.99–1.05)	0.088		
Sex (Rf = male)	0.9 (0.57–1.42)	0.657		
ECOG (Rf = 0–1)	0.3 (0.19–0.47)	**< 0.001**	0.36 (0.18–0.57)	***P*** **<** **0.001**
Hypertension (Rf = yes)	0.91 (0.56–1.46)	0.690		
Hyperglycemia (Rf = yes)	0.63 (0.34–1.16)	0.139		
**Laboratory biomedical features**				
HDL	0.84 (0.48–1.48)	0.556		
LDL	0.88 (0.68–1.15)	0.361		
Cholesterol	0.91 (0.74–1.11)	0.350		
Triglyceride	1.01 (0.82–1.25)	0.924		
Albumin	0.95 (0.92–0.99)	**0.014**	0.98 (0.93–1.04)	0.483
**Anthrometric measures**				
**BMI categories, (kg/m** ^2^ **)**				
Underweight	4.57 (2.39–8.76)	**< 0.001**	2.58 (1.21–5.50)	0.014
Overweight	0.4 (0.22–0.73)	**0.003**	0.55 (0.26–1.15)	0.110
**Adipose and muscle tissue distribution**				
SFI (Rf: SFI < 26.4)	2.1 (1.32–3.32)	**0.002**	1.82 (1.02–3.33)	**0.049**
PFI (Rf: PFI < 38.66)	1.4 (0.82–2.42)	0.221		
**Sarcopenia**	1.78 (1.11–2.85)	**0.016**	1.21 (0.69–2.14)	0.504
**Immuno-nutritional scores**				
PNI (Rf: PNI < 45)	1.87 (1.15–3.02)	**0.011**	1.04 (0.52–2.07)	0.903
GPS (Rf: GPS = 1–2)	1.24 (0.78–1.97)	0.361		
CAR (Rf: CAR < 1.44)	0.33 (0.20–0.55)	**0.001**	0.34 (0.18–0.63)	**0.001**
**Inflammation indexes**				
NLR (RF:NLR < 3.8)	0.91 (0.58–1.45)	0.704		
SII (Rf: SII < 296.31)	1.57 (0.94–2.64)	0.087		
PLR (Rf: PLR < 169.1)	1.04 (0.65–1.66)	0.872		

Abbreviation: SFI, subcutaneous fat index; PFI, Pericardial Fat Index; SMI, Skeletal muscle index; PNI, the Prognostic Nutritional Index; GPS, the Glasgow Prognostic Score; CAR, C-reactive protein-to-albumin ratio; SII, systemic immune-inflammation index; PLR, platelet-to-lymphocyte ratio; NLR, neutrophil-to-lymphocyte ratio; HR, hazard ratio; CI, confidence interval.

### 3.4 Systemic inflammation parameters with body composition

Through logistic regression analyses, we investigated the relationship between body composition markers (PFI, SFI, SMI) and systemic immune-nutritional scores (CAR, PNI, SII, GPS, NLR, PLR), while adjusting for age, sex, PNI, SII, PLR, NLR, CAR, GPS, tumor histology, ECOG performance status, smoking status, radiation treatment sites, BMI, hypertension, hyperglycemia, albumin levels, triglycerides, cholesterol, HDL, and LDL. Detailed results are presented in Table 5, Supplementary Tables S2, S3. The results indicated that among all systemic immune-nutritional scores, only PNI was associated with body composition biomarkers, emerging as an independent risk factor for all three markers, including SFI [odds ratio (OR) = 2.77, 95% CI 1.01–7.99, *P* = 0.042] (Table 5), SMI (OR = 1.98, 95% CI 1.66–6.17, *P* = 0.026) (Supplementary Table S2), and PFI (OR = 1.41, 95% CI 1.28–4.37, *P* = 0.030) (Supplementary Table S3).

**Table 5:** Univariate and multivariate logistic analyses on immuno-nutritional scores and SFI.

**Variables**	**Univariate analysis**		**Multivariate analysis**	
	**OR**	***P*-value**	**OR**	***P-*value**
**Demographic features**				
Age	0.86 (0.38–1.93)	0.717		
Sex	0.66 (0.30–1.47)	0.314		
ECOG	0.64 (0.28–1.42)	0.268		
Hypertension	1.73 (0.76–3.96)	0.191		
Hyperglycemia	0.48 (0.16–1.31)	0.168		
**Smoking status**				
**Current_somker**				
Non-smoker	0.54 (0.15–1.84)	0.329		
Past-smoker	0.62 (0.23–1.67)	0.344		
**Radiotherapy site**				
**Bone**				
Brain	0.41 (0.14–1.16)	0.096	0.31 (0.09–1.01)	0.048
Lung	0.10 (0.01–0.75)	0.052	0.05 (0.00–0.44)	0.017
Others	0.73 (0.18–2.93)			
**Laboratory biomedical features**				
HDL	0.67 (0.23–1.69)	0.421		
LDL	0.65 (0.38–1.06)	0.092		
Cholesterol	0.71 (0.47–1.03)	0.081		
Triglyceride	0.88 (0.58–1.28)	0.883		
Albumin	0.99 (0.93–1.01)	0.593		
**BMI categories, (kg/m**^2^)				
Normal				
Overweight	0.43 (0.15–1.13)	0.097	0.33 (0.09–1.00)	0.06
Underweight	1.81 (0.52–6.73)	0.351	0.87 (0.20–3.80)	0.856
**Immuno-nutritional scores**				
PNI (PNI < 45)	3.19 (1.35–7.75)	0.009	2.77 (1.01–7.99)	**0.042**
SII (SII < 296.31)	0.68 (0.25–1.74)	0.434		
PLR (PLR < 169.1)	0.51 (0.23–1.15)	0.107		
NLR (NLR < 3.8)	0.83 (0.37–1.86)	0.647		
CAR (CAR < 1.44)	0.64 (0.26–1.57)	0.327		
GPS (GPS = 1–2)	1.95 (0.87–4.44)	0.107		

Statistical significance was set at *P* < 0.05. The expected count should be < 5 to follow the Fisher's exact test results. Abbreviation: PNI, the Prognostic Nutritional Index; SII, systemic immune-inflammation index; PLR, platelet-to-lymphocyte ratio; NLR, neutrophil-to-lymphocyte ratio; CAR, C-reactive protein-to-albumin ratio; GPS, the Glasgow Prognostic Score; CI, confidence interval; OR, odds ratio.

### 3.5 Body composition and prognosis nutritional index are predictors for PFS and OS in oligometastatic NSCLC receiving radiotherapy

**Figure 4:**
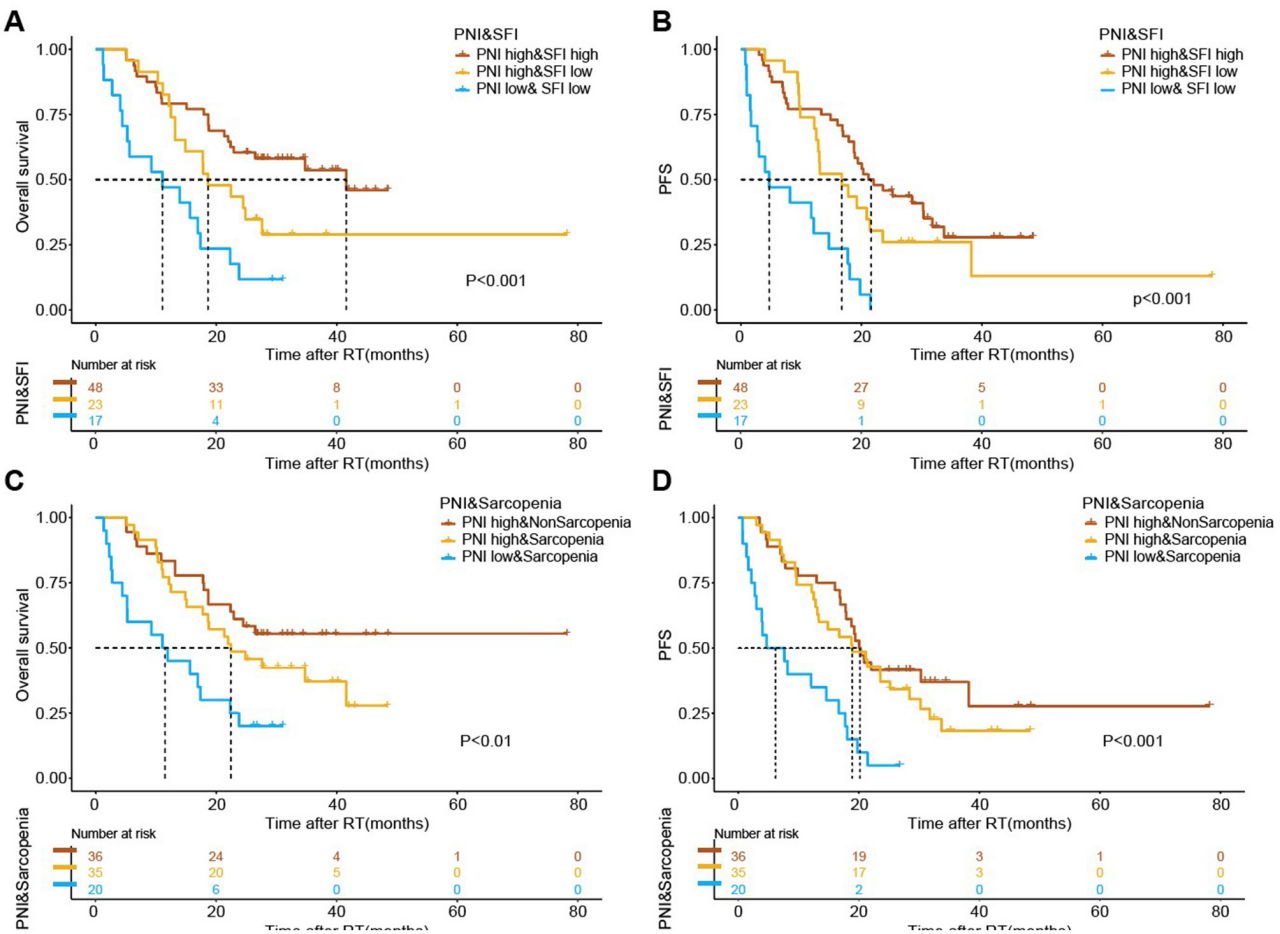
The association of combined body composition and immuno-nutritional scores with survival outcomes following radiotherapy. Kaplan-Meier estimates for median overall survival (OS) and progression-free survival (PFS) stratified by the combination of Prognostic Nutritional Index (PNI) and Subcutaneous Fat Index (SFI) **(A, B)**, and PNI and sarcopenia **(C, D)**. The respective cutoff for each parameter and median survival in months are depicted above each graph. The *P*-value from the Mantel–Cox log-rank test is indicated in the graph inset.

To further elucidate the impact of these parameters on survival outcomes, we conducted K-M analyses by combining PNI (high vs. low) with SFI (high vs. low) and sarcopenia (sarcopenia vs. non-sarcopenia). The results indicated significant prognostic differences among the various groups (Figure 4). The group with high PNI and high SFI exhibited markedly improved OS and PFS, as well as the one with high PNI and without sarcopenia (*P* < 0.001). Therefore, the combination of elevated subcutaneous fat or muscle mass with high PNI emerges as a favorable prognostic indicator for patients undergoing radiotherapy.

## 4 Discussion

This study represented the first investigation into the independent and combined impact of body composition and immune-nutritional status on the prognosis of oligometastatic NSCLC patients undergoing radiotherapy. Our findings underscored the significant associations among sarcopenia, adiposity indices, and various inflammatory markers. BMI, ECOG performance status, PFI, and CAR emerged as critical prognostic factors for OS, and BMI, ECOG performance status, SFI, and CAR were identified as key predictors for PFS. These results highlighted the pivotal role of body composition in influencing treatment response and underscored the necessity for future translational research to elucidate potential immune-metabolic mechanisms.

Inflammation has been recognized as a crucial prognostic determinant in cancers receiving radiotherapy, such as melanoma and renal cell carcinoma (28, 29). The inflammatory status discussed here needs to be distinguished from the concept of tumor inflammatory microenvironment. Our results indicated that the PNI, which reflects nutritional status and immune balance, serves as a robust predictive indicator of survival in these patients, with higher PNI values correlating with improved outcomes. This study provided oncologists with compelling evidence that comprehensive nutritional assessment is vital for optimizing the care of patients with oligometastatic NSCLC.

Our analyses also revealed the significant prognostic value of evaluating muscle-related body composition. Elevated muscle mass indicators, such as SMI, correlated positively with favorable survival outcomes. In the context of cancer, systemic inflammatory responses markedly contribute to muscle depletion through mechanisms involving increased oxidative stress and mitochondrial dysfunction, which adversely affect skeletal muscle cells within the tumor microenvironment (30). Consequently, the decline in muscle mass disrupts the secretion of antitumor factors (myokines), ultimately leading to poorer prognoses. These physiological alterations are expected in malnourished cancer patients, with SMI acting as a crucial prognostic marker (31).

Adipose tissue, encompassing subcutaneous and visceral fat, also plays a vital role in patients with NSCLC (27). Our results indicated that more subcutaneous adipose tissue is beneficial for PFS in these individuals. Similarly, an increase in pericardial fat content correlates with improved prognosis, showing significant associations with visceral fat. We quantified skeletal muscle and fat, establishing critical thresholds for sarcopenia, SFI and PFI reductions. Obesity and elevated PFI were found to be independently associated with OS, after adjusting for body composition variables. In contrast, sarcopenia did not independently impact OS, potentially due to the heightened energy requirements of cancer patients, with fat serving as a key energy source (32). In cancer patients, fat depletion often precedes muscle wasting, a pattern also observed in gastrointestinal cancers (33). This phenomenon is characterized by enhanced lipolysis and reduced adipocyte volume, leading to depleted energy reserves and disrupted homeostasis, which correlates with diminished survival times (34). Therefore, we attribute the obesity paradox in patients with oligometastatic NSCLC more to adipose tissue than to skeletal muscle.

**Figure 5:**
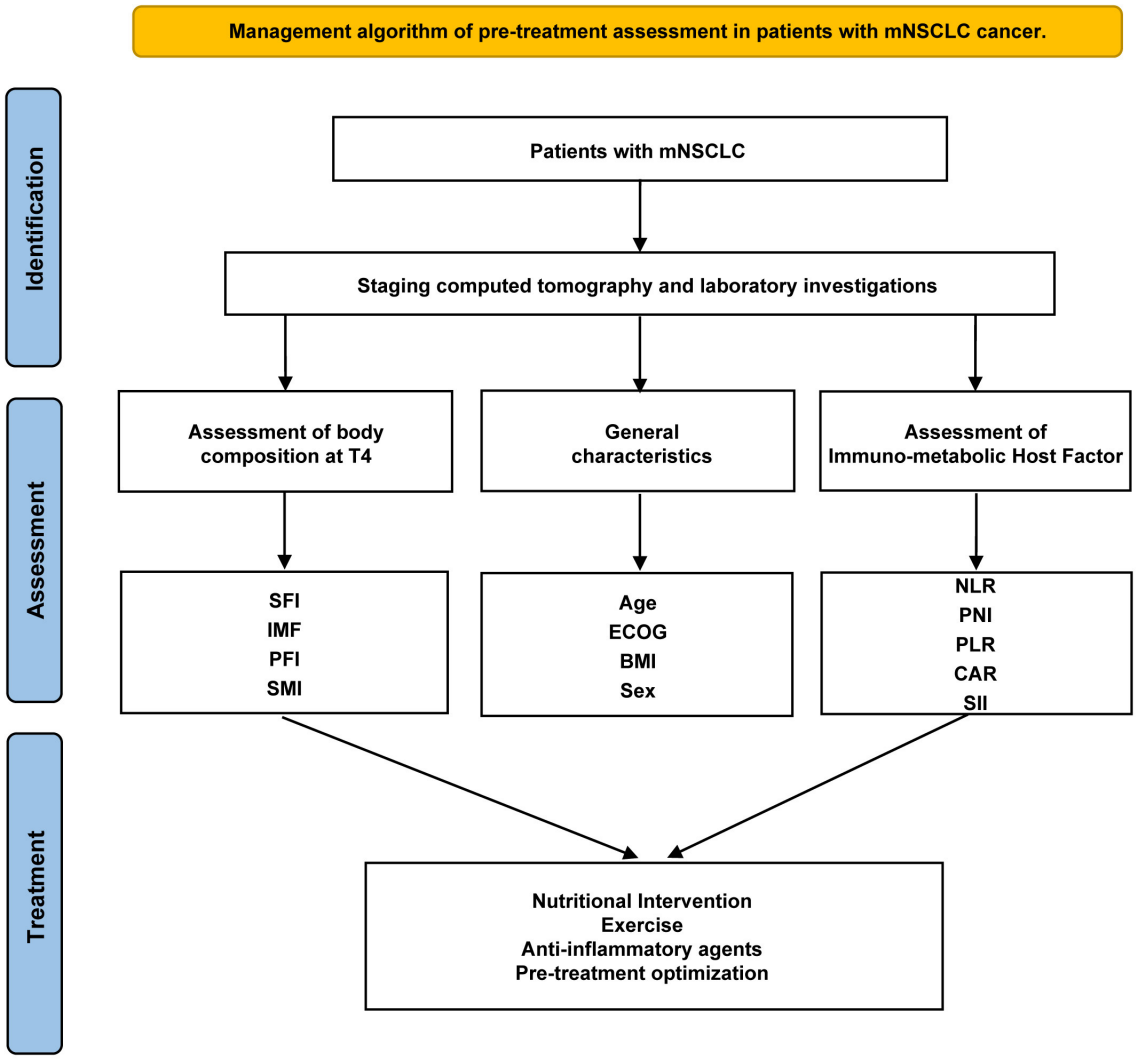
Management algorithm of pre-treatment assessment in patients with mNSCLC. SFI, subcutaneous fat index; PFI, Pericardial Fat Index; SMI, Skeletal muscle index; PNI, the Prognostic Nutritional Index; GPS, the Glasgow Prognostic Score; CAR, C-reactive protein-to-albumin ratio; SII, systemic immune-inflammation index; PLR, platelet-to-lymphocyte ratio; NLR, neutrophil-to-lymphocyte ratio.

However, relying solely on these parameters may not comprehensively determine treatment responses or prognosis. Other disease-specific and host intrinsic factors must also be considered. While developing a universal tool to guide treatment for all patients remains challenging, thorough assessment, combined with early palliative interventions, may constitute the most effective strategy prior to initiating treatment. Consequently, exploring a multimodal approach that integrates nutritional, exercise, and anti-inflammatory interventions with cancer therapies may enhance therapeutic efficacy (Figure 5).

There are several limitations to our study. First, selection bias is unavoidable due to the retrospective and single-center design. Second, we did not assess the impact of changes in body composition post-radiotherapy or the effects of neoadjuvant and adjuvant chemotherapy. Third, considering clinical feasibility, we measured body composition at the Th4 level rather than the standard L3 level, which may affect comparability. Future directions include prospective data collection to further validate our findings and the necessity for multicenter studies to confirm these results. If the preliminary findings of this study are substantiated in future prospective investigations, they will provide significant guidance for clinical management. Additionally, forthcoming research should investigate the impact of physical activity and diet on outcomes. In particular, a better understanding is needed regarding the impact of diet in modulating gut microbiome composition and its potential effects on immune responses is warranted. Increasing evidence indicates the role of the gut microbiome in immune modulation, and elucidating the interplay between diet and the gut microbiome holds clinical significance for enhancing the efficacy of radiotherapy and other cancer treatments (35). Therefore, future studies must also focus on the relationship between diet, the gut microbiome, and cancer treatment responses to develop more personalized and effective therapeutic strategies.

## 5 Conclusion

In summary, our study underscores the significance of immune-nutritional scores, and body composition assessed via baseline CT as vital prognostic indicators for oligometastatic NSCLC patients. The findings imply that implementing multimodal clinical management strategies aimed at mitigating the loss of subcutaneous adiposity could enhance the life quality and prognosis of patients with advanced NSCLC. The underlying mechanisms and validation of these results warrant further investigation.

## Data Availability

The original contributions presented in the study are included in the article/Supplementary material, further inquiries can be directed to the corresponding authors.
