# Supplementary material for: Prognostic impact of body composition and immune-nutritional status in oligometastatic NSCLC patients receiving radiotherapy
Source: Front Nutr. 2025 Jul 1;12:1588391. doi: 10.3389/fnut.2025.1588391 (PMC12261918; doi:10.3389/fnut.2025.1588391)
Supplement: Supplementary file 1 [file Table_1.docx]

**Table S1.** The definition and cutoff value of inmmuno-nutritional scores

| **Variables** | **Cutoff value** | **definition** |
| --- | --- | --- |
| SII |  | Platelet count (× 10^9^/L) × neutrophil count (×10^9^ /L) / lymphocyte count (×10^9^ /L) |
| NLR |  | Neutrophil count (×10^9^ /L): lymphocyte count (× 10^9^ /L) |
| PLR |  | Platelet count (×10^9^ /L): lymphocyte count (× 10^9^ /L) |
| CAR |  | CRP [mg/L] / albumin [g/dL] |
| PNI |  | Albumin+5 × total lymphocyte count (×10^9^ /L) |
| GPS |  |  |
|  | 0 | CRP ≤ 10 [mg/L] |
|  | 1 | CRP > 10 [mg/L] + albumin > 3.5 [g/dL] |
|  | 2 | CRP > 10 [mg/L] + albumin < 3.5 [g/dL] |

Abbreviations:SII,systemic immune-inflammation index; NLR, neutrophil-to-lymphocyte ratio; PLR, platelet-to-lymphocyte ratio; CAR,C-reactive protein-to-albumin ratio; PNI,the Prognostic Nutritional Index; GPS,the Glasgow Prognostic Score.

**Table S2.** Univariate and multivariate logistic analyses on immuno-nutritional scores and SMI

| Variables | **Univariate analysis** | | **Multivariate analysis** | |
| --- | --- | --- | --- | --- |
|  | **OR** | ***P* value** | **OR** | ***P* value** |
| **Demographic features**  Age  Sex  ECOG  Hypertension  Hyperglycemia  Smoking status  Current_somker  Non-smoker  Past-smoker  Radiotherapy site  Bone  Brain  Lung  Others  **Laboratory biomedical features**  HDL  LDL  Cholesterol  Triglyceride  Albumin  **BMI categories, (kg/m2)**  Normal  Overweight  Underweight  **Immuno-nutritional scores**  PNI (PNI<45)  SII (SII<296.31)  PLR (PLR<169.1)  NLR (NLR<3.8)  CAR (CAR<1.44)  GPS (GPS=1-2) | **2.29 (1.02-5.28)**  0.53 (0.24-1.16)  **0.34 (0.15-0.77)**  0.95 (0.42-2.14)  0.78 (0.30-2.02)  0.56 (0.15-2.04)  0.33 (0.11-0.92)  1.71 (0.61-4.92)  1.85 (0.35-11.23  0.62 (0.14-2.51)  1.34 (0.55-3.63)  1.15 (0.72-1.85)  1.15 (0.81-1.66)  0.87 (0.59-1.26)  1.01 (0.99-1.06)  0.71 (0.29-1.75)  2.47 (0.66-11.94)  **1.91 (1.81-4.66)** 1.90 (0.75-5.16)  1.17 (0.53-2.61)  1.40 (0.63-3.11)  0.79 (0.32-1.90)  0.61 (0.27-1.36) | **0.048**  0.113  **0.011**  0.897  0.602  0.384  0.041  0.309  0.475  0.504  0.532  0.567  0.443  0.452  0.529  0.460  0.205  **0.044**  0.189  0.695  0.406  0.596  0.229 | **2.59 (1.06-6.66)**  **0.36 (0.14-0.88)**  1.38 (0.28-6.95)  1.05 (0.20-5.65)  **1.98 (1.66-6.17)** | **0.041**  **0.027**  0.694  0.953  **0.026** |

Statistical significance was set at P < 0.05. The expected count should be <5 to follow the Fisher’s exact test results. PNI,the Prognostic Nutritional Index; SII, systemic immune-inflammation index; PLR, platelet-to-lymphocyte ratio; NLR, neutrophil-to-lymphocyte ratio; CAR,C-reactive protein-to-albumin ratio; GPS,the Glasgow Prognostic Score; CI, confidence interval; OR, odds ratio.

**Table S3.** Univariate and multivariate logistic analyses on immuno-nutritional scores and PFI

| Variables | **Univariate analysis** | | **Multivariate analysis** | |
| --- | --- | --- | --- | --- |
|  | **OR** | ***P* value** | **OR** | ***P* value** |
| **Demographic features**  Age  Sex  ECOG  Hypertension  Hyperglycemia  Smoking status  Current_somker  Non-smoker  Past-smoker  Radiotherapy site  Bone  Brain  Lung  Others  **Laboratory biomedical features**  HDL  LDL  Cholesterol  Triglyceride  Albumin  **BMI categories, (kg/m2)**  Normal  Overweight  Underweight  **Immuno-nutritional scores**  PNI (PNI<45)  SII (SII<296.31)  PLR (PLR<169.1)  NLR (NLR<3.8)  CAR (CAR<1.44)  GPS (GPS=1-2) | 1.45 (0.58-3.79)  1.23 (0.50-3.04)  0.65 (0.25-1.62)  2.30 (0.87-6.87)  0.51 (0.19-1.44)  0.67 (0.14-2.96)  0.56 (0.15-1.78)    1.00 (0.29-3.11)  1.07 (0.17-8.96)  1.31 (0.26-7.53)  1.41 (0.51-5.03)  0.84 (0.50-1.43)  0.86 (0).58-1.28  0.76 (0.51-1.14)  1.02 (0.99-1.10)  0.43 (0.16-1.18)  0.48 (0.13-2.04)  **1.51 (1.55-3.78)**  0.60 (0.22-1.68)  0.37 (0.12-0.98)  0.43 (0.15-1.11  1.71 (0.63-4.44)  0.68 (0.28-1.69) | 0.432  0.650  0.369  0.110  0.191  0.592  0.355  0.994  0.943  0.746  0.548  0.519  0.438  0.168  0.559  0.099  0.289  0.043  0.316  0.056  0.092  0.278  0.402 | 0.39 (0.14-1.12)  0.37 (0.08-1.68)  **1.41 (1.28-4.37)**  0.50 (0.14-1.61)  0.50 (0.14-1.60) | 0.078  0.177  **0.030**  0.259  0.257 |

Statistical significance was set at P < 0.05. The expected count should be <5 to follow the Fisher’s exact test results. PNI,the Prognostic Nutritional Index; SII, systemic immune-inflammation index; PLR, platelet-to-lymphocyte ratio; NLR, neutrophil-to-lymphocyte ratio; CAR,C-reactive protein-to-albumin ratio; GPS,the Glasgow Prognostic Score; CI, confidence interval; OR, odds ratio.
